# Posterior Deep Infiltrating Endometriotic Nodules: Operative Considerations according to Lesion Size, Location, and Geometry, during One's Learning Curve

**DOI:** 10.1155/2014/853902

**Published:** 2014-01-20

**Authors:** Athanasios Protopapas, Georgios Giannoulis, Ioannis Chatzipapas, Stavros Athanasiou, Themistoklis Grigoriadis, Dimitrios Haidopoulos, Dimitrios Loutradis, Aris Antsaklis

**Affiliations:** 1st Department of Obstetrics and Gynecology, University of Athens, “Alexandra” Hospital, 3 Aisopou Street, Marousi, 15122 Athens, Greece

## Abstract

We conducted this prospective cohort study to standardize our laparoscopic technique of excision of posterior deep infiltrating endometriosis (DIE) nodules, according to their size, location, and geometry, including 36 patients who were grouped, according to principal pelvic expansion of the nodule, into groups with central (group 1) and lateral (group 2) lesions, and according to nodule size, into ≤2 cm (group A) and >2 cm (group B) lesions, respectively. In cases of group 1 the following operative steps were more frequently performed compared to those of group 2: suspension of the rectosigmoid, colpectomy, and placement of bowel wall reinforcement sutures. The opposite was true regarding suspension of the adnexa, systematic ureteric dissection, and removal of the diseased pelvic peritoneum. When grouping patients according to nodule size, almost all of the examined parameters were more frequently applied to patients of group B: adnexal suspension, suspension of the rectosigmoid, systematic ureteric dissection, division of uterine vein, colpectomy, and placement of bowel wall reinforcement sutures. Nodule size was the single most important determinant of duration of surgery. In conclusion, during the building-up of one's learning curve of laparoscopic excision of posterior DIE nodules, technique standardization is very important to avoid complications.

## 1. Introduction

Deep infiltrating endometriosis (DIE) is a particular form of endometriosis that extends >5 mm under the peritoneal surface [1]. These lesions develop in the form of retroperitoneal nodules that consist histologically of endometrial epithelium and stroma, surrounded by muscular hyperplasia and fibrosis [2]. DIE nodules are rich in nerve fibers [3] and are commonly associated with severe cyclic or acyclic pelvic pain such as dysmenorrhea, deep dyspareunia, and nonmenstrual pain and organ-specific symptoms related to bladder or intestinal dysfunction (dyschezia, constipation, diarrhea, rectal bleeding, frequency of micturition, and hematuria) [4–6].

Radical surgical exeresis of DIE lesions is the mainstay of treatment for this form of endometriosis. Medical therapies may temporarily alleviate painful symptoms, but recurrence rates after their discontinuation are high [7, 8]. Furthermore, performing inadequate primary surgery not only results in disease progression with persistence or aggravation of painful symptoms but also renders any future procedure difficult and potentially dangerous [9, 10].

DIE nodules represent a real operative challenge due to common involvement of vital retroperitoneal structures (ureter, bowel, vessels, and nerves). Laparoscopy appears to be the ideal tool to perform such surgery, offering the advantages of magnification, accurate hemostasis, precise dissection, and careful handling of delicate tissue. Nevertheless, laparoscopic management of retroperitoneal endometriosis should not be undertaken by inexperienced operators and thorough knowledge of pelvic retroperitoneal anatomy is a prerequisite for radical and uncomplicated removal of DIE nodules.

Posterior nodules represent the commonest form of deep infiltrating endometriosis [11, 12]. Their radical exeresis may require extensive retroperitoneal surgery which may include resection of the uterosacral ligaments (USL), partial colpectomy, and resection of rectal disease. A multidisciplinary surgical approach may be necessary involving a urologist or a bowel surgeon with expertise in advanced laparoscopic surgery to successfully conclude the procedure [13, 14].

Although laparoscopic excision of large DIE nodules may become a quite unpredictable operation in terms of following distinct and timely operative steps, an effort should be made during one's learning curve to develop a standardized way of approaching such lesions. We conducted this study in an effort to standardize our laparoscopic technique of excision of posterior DIE according to the size, location, and geometry of the lesion, attempting at the same time to develop a rough guide for relatively inexperienced surgeons embarking on the surgical management of posterior DIE nodules.

## 2. Materials and Methods

From September 2008 to July 2011 we recruited for this study 40 consecutive patients with a prospective diagnosis of DIE based on their symptoms, clinical examination, and preoperative workup. Institutional review board approval was obtained for this study, and a detailed informed consent was signed by all women. All patients were initially referred to our institution for chronic pelvic pain (CPP), infertility, or the presence of endometriotic ovarian cysts and, in the process of their evaluation, were found with a pelvic nodule suggestive of DIE. All women completed a detailed pain, sexual function, and quality of life (QoL) questionnaire [15] and underwent a thorough pelvic examination (i.e., vaginal and rectal). A transvaginal (TVS) and/or transrectal (TRS) ultrasound scan was performed to assess the adnexa and uterus and to estimate the size and exact position of the nodule in relation to the wall of the rectosigmoid and the pelvic sidewall. Magnetic resonance imaging (MRI) of the pelvis was also performed in cases with dubious findings and in cases with large lesions, to obtain more detailed information on the exact geometry of the nodule. For posterolateral lesions an upper abdominal scan was performed to investigate the possibility of ureteral involvement resulting in hydronephrosis by the disease process. Cases with considerable disturbances of bowel function were submitted to rectosigmoidoscopy. Preoperative CA-125 and CA-19-9 levels were also measured in all cases.

All patients were scheduled for a laparoscopic procedure outside their days of menstruation. No medical ovarian suppression such as GnRH analogs was administered preoperatively. Peritoneal entry and the pneumoperitoneum were established following a standardized transumbilical blind technique using the Veress needle. In cases with a history of previous gynecological or lower abdominal surgery, initial entry was achieved through the left subcostal area, followed by placement of a 10 mm trocar for the optic through the umbilicus. Three 5 mm accessory trocars for the laparoscopic instruments were used, placed in a standardized fashion above the pubic hairline [14].

During the diagnostic phase of the procedure, pelvic endometriosis was staged according to the revised American Fertility Society (rAFS) classification [16] and DIE according to the ENZIAN classification [17]. The laparoscopic procedure to treat ovarian and superficial endometriosis and to perform pelvic adhesiolysis followed certain operative rules in a timely fashion and was common for all types of DIE nodules. Excision of DIE nodules followed completion of these initial operative steps. Both posterolateral and central nodules were approached step by step, recording each operative step according to its absolute necessity for the procedure to advance smoothly. Recorded technical parameters included the necessity for ovarian and/or sigmoid suspension, systematic ureteral dissection, removal of parts of the pelvic peritoneum, division of large uterine vascular branches, partial colpectomy, and rectosigmoid reinforcement suturing. Other recorded parameters included intraoperative blood loss, length of the procedure, and hospitalization times. All excised specimens were sent for histology. A prerequisite for a case to be included in this study was the histologic confirmation of the presence of endometriosis (epithelium and stroma) in the excised DIE nodule.

### 2.1. Operative Technique

In all cases the operation began with adhesiolysis commencing at the level of the pelvic brim, following a direction from top to bottom and laterally to medially. Congenital sigmoid adhesions were routinely divided in order to facilitate rectosigmoid suspension, should this appeared necessary during the procedure. Both adnexae were mobilized with division of their adhesions with the ovarian fossa, and careful hemostasis was performed to avoid contamination of the operative field. In cases with endometriomas the cyst was evacuated and the cavity thoroughly washed for the same reason. Ovarian surgery, when necessary, was left to follow excision of the DIE.

Following these common preparatory steps the DIE nodule-harboring area to be excised was selected. Selection was facilitated through careful intraoperative digital transvaginal and/or transrectal palpation performed by the surgeon. Suspension of either the adnexa or the rectosigmoid was decided at this point according to quality of the operative field obtained without the need to systematically occupy an instrument as a retractor. The ovarian and rectosigmoid suspension techniques used have already been described by others [14].

**Figure 1:**
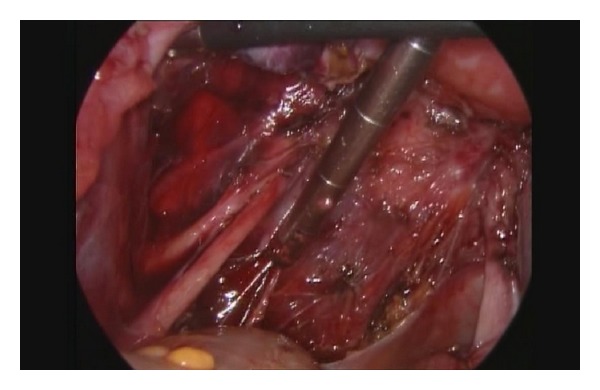
Complete dissection of the ureter, with sparing of the uterine vessels in a case with a posterolateral E2b DIE nodule and grossly involved pelvic peritoneum.

**Figure 2:**
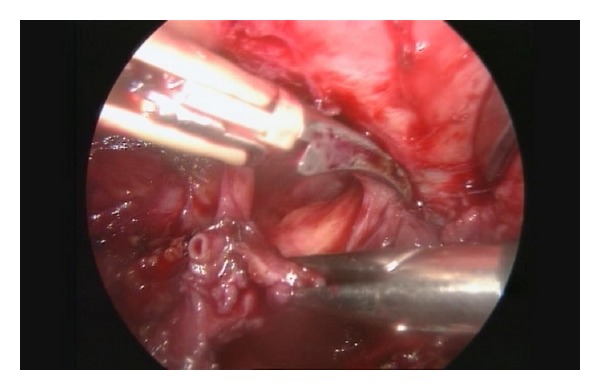
Division of the uterine vein in a case with an E3b DIE nodule.

In cases with posterolateral nodules the retroperitoneal space was entered through a small peritoneal incision at a healthy area as close as possible to the nodule. The incision was enlarged along the periphery of the diseased peritoneum of the ovarian fossa, and the peritoneal area to be excised was selected. At this point it was also decided whether systematic ureteral dissection (Figure 1) appeared necessary. The decision depended on the following parameters: the degree of lateral fibrosis, the degree of peritoneal involvement, the degree of uterosacral ligament involvement, and the difficulty to recognize relevant retroperitoneal structures. Ureteral dissection was advanced distally towards the ureter's point of crossing with the uterine artery. Similarly, the decision to bypass, fully dissect, or sacrifice large uterine vascular branches (Figure 2) was made at that point, according to degree of their involvement by the DIE nodule. The uterosacral ligament was subsequently divided at the point of its insertion to the uterine cervix. The 2nd assistant's vaginal finger served as a guide to limit the incision to a healthy plane in relation to the vaginal fornix. At this point the decision to perform or not partial colpectomy was made. To achieve radical excision of the nodule both medial and lateral ipsilateral rectovaginal spaces were developed in all cases with posterolateral nodules. In cases with rectal wall involvement the shaving technique was always attempted to excise the nodule. Following its' removal a decision was made whether or not to reinforce the bowel wall with horizontal interrupted absorbable sutures.

**Figure 3:**
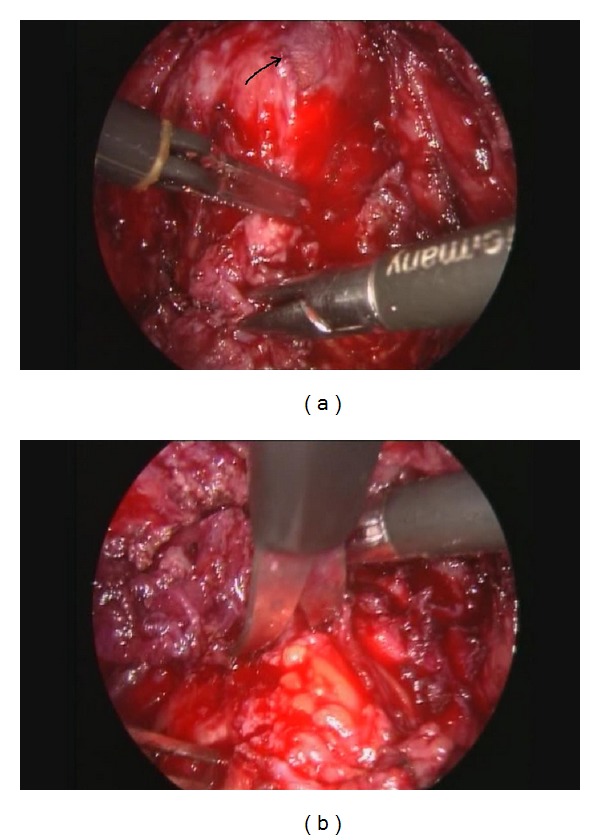
(a) Shaving technique to remove an E2a–E2c legion with opening the vagina (arrow). (b) Shaving technique to remove an E3c legion from the rectosigmoid.

In cases with predominantly central lesions, the technique was modified according to presence of unilateral or bilateral involvement of the uterosacral ligaments. In the case of purely central nodules, opening of the retroperitoneal space was achieved from the least involved side by incising the peritoneum medially to the ipsilateral uterosacral ligament. The ipsilateral medial rectovaginal space was thoroughly dissected below the level of the lowest limit of the nodule. Similarly, the contralateral space was developed, as far down as possible. At this point a sponge was inserted in the posterior vaginal fornix and another in the rectum, to assist in the identification of the plane between these two organs. In cases without a recognizable plane, possibly suggestive of a nodule predominantly attached to the rectosigmoid, the procedure was advanced by incising the peritoneum lying below the uterine torus laterally to medially. At this point a decision was made to perform or not partial colpectomy depending on the degree of vaginal induration caused by the nodule. Although preoperative findings during examination under anesthesia were taken into account, colpectomy was by no means a predetermined operative step. Following colpotomy, the vaginal lesion was circumscribed by distally incising the vaginal mucosa, and the lesion was left attached to the rectosigmoid. The same applied to cases managed without colpotomy. In such patients multiple intraoperative vaginal examinations assisted in identifying the correct plane for vaginal dissection. Following complete mobilization of the nodule from the vagina with or without colpotomy the nodule was gradually detached from the rectosigmoid using the shaving technique (Figures 3(a) and 3(b)). The decision to reinforce the bowel wall with horizontal sutures depended on the degree of muscular wall involvement.

In cases with central nodules bearing a lateral component which involved significantly the uterosacral ligament(s) the technique was modified according to geometry of the lateral extension. Similar operative steps and timely decisions were made as those described above, in order to optimally approach the DIE nodule. Occasionally, the order of decision making was changed and the operative steps were adapted to serve better the individual patient.

Discoid rectal excision or rectosigmoidectomy, where necessary, was undertaken by a general surgeon with relevant expertise after the preparation of the operative field as described above by the laparoscopic gynecologic team. A trained urologist was also involved in cases where ureteral surgery other than ureterolysis was necessary.

Follow-up examinations were scheduled at six monthly intervals for two years. During each appointment patients complete the pain, sexual function, and QoL questionnaire and receive a vaginal and rectal examination. TVS and/or TRS are performed in case of abnormal findings.

### 2.2. Statistical Analysis

Fisher's exact test was used to compare differences between frequencies of adnexal and rectosigmoid suspension, systematic ureteral dissection, removal of parts of the pelvic peritoneum, division of large uterine vascular branches, partial colpectomy, rectosigmoid reinforcement suturing, and rates of intraoperative and postoperative complications, according to location (predominantly central versus predominantly lateral) and size (≤2 cm versus >2 cm) of the DIE nodule. Student's 2-independent samples *t*-test was used to compare operative parameters expressed in numerical values such as intraoperative blood loss, operation times, and duration of hospitalization between groups. Significance level was set to *P* < 0.05.

## 3. Results

Patient characteristics are presented in Table 1. Four patients were excluded from further analysis. Of these, one was found with complete obliteration of the Pouch of Douglas by adhesions, but without real deeper involvement. Three further patients were laparoconverted. The first case was a 46-year-old patient, with a poorly mobile voluminous adenomyotic uterus combined with a 4 cm central DIE nodule, who was managed with modified radical hysterectomy. The second case had left ureteral obstruction with hydronephrosis due to a 3 cm nodule in whom excision of the involved segment and end-to-end ureteral reanastomosis was performed. The last case was a 29-year-old patient with a 3 cm hemorrhagic lesion invading the vaginal mucosa that were surprisingly found at frozen section to correspond to a primary serous papillary carcinoma of the peritoneum.

**Figure 4:**
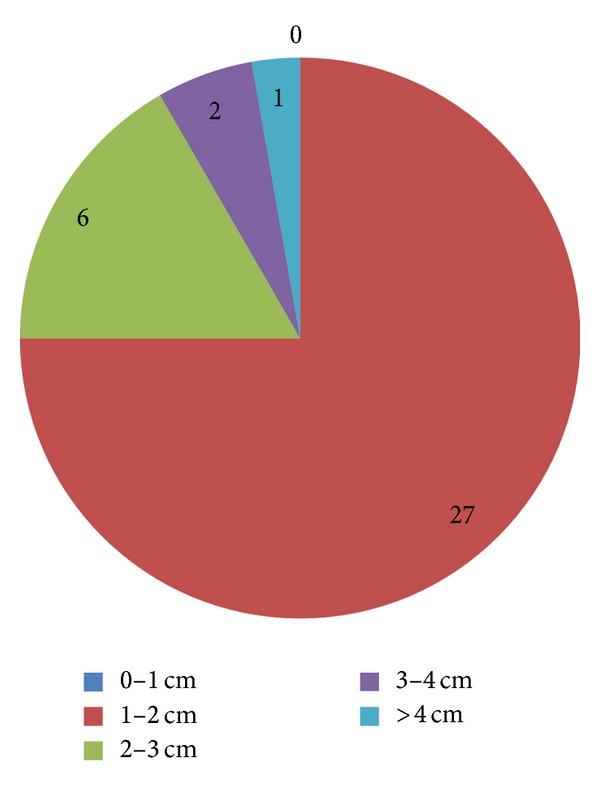
Distribution of our cases according to DIE nodule size.

**Figure 5:**
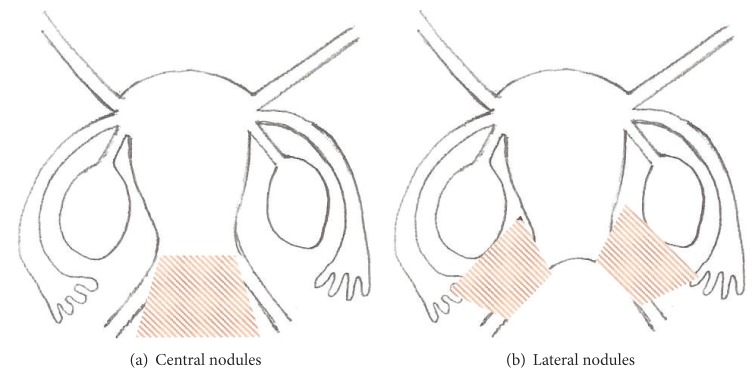
Grouping of our patients according to location of the DIE nodules.

In Table 2 our cases are presented according to their ENZIAN classification taking into account the two most involved compartments.  Figure 4 presents distribution of our cases according to size of the DIE nodule. For purposes of more meaningful analysis of our technical considerations, we grouped together (group 1, Figure 5(a)) nodules spreading along compartments a and c (predominantly central nodules). Spread along the horizontal plane b constituted the group with predominantly lateral nodules (group 2, Figure 5(b)).

Suspension of the adnexa to facilitate exposure of the ovarian fossa and ipsilateral uterosacral ligament was more frequently necessary in cases of group 2 (91.7% versus 58.3%, resp., *P* = 0.029). On the contrary, suspension of the rectosigmoid was considered necessary for the procedure to advance smoothly in the majority of group 1 patients (66.7% versus 12.5%, resp., *P* = 0.002). The peritoneum of the ovarian fossa was the initial point of entry to the retroperitoneal space in all cases with lateral nodules compared with only 2 cases of the group with central nodules in whom the peritoneum overlying the lateral rectovaginal space was opened (100.0% versus 16.6%, resp., *P* < 0.001).

The ureter was systematically dissected down to its' crossing with the uterine artery in the majority of group 2 cases (85.7% versus 41.7%, resp., *P* = 0.007). Similarly, the diseased and fibrotic part of the peritoneum of the ovarian fossa was removed in all patients with lateral nodules, compared with less than half of patients with central nodules (100.0% versus 50.0%, resp., *P* < 0.001). During the process of liberating the nodule from its retroperitoneal attachments it was considered necessary to sacrifice the uterine vein in 3 of group 2 cases compared with none of group 1 (12.5%, versus 0.0%, resp., NS). On the contrary, colpectomy was considered necessary in 3 cases with central nodules compared with none of those with lateral lesions (25.0% versus 0.0%, resp., *P* = 0.031).

The DIE nodule was firmly attached to the wall of the rectosigmoid in 5/12 (41.7%) of group 1 and in 7/24 (33.3%) of group 2 cases, respectively (Table 2). The shaving technique was performed in all but one patient. This last case corresponded to an E4c lesion according to the ENZIAN classification and was managed with rectosigmoidectomy of the affected segment and end-to-end reanastomosis. Bowel wall reinforcement sutures were considered necessary in 3 of group 1 cases compared to none of group 2 (37.5% versus 0.0%, resp., *P* = 0.011).  Table 3 summarizes the above findings.

When grouping patients according to nodule size (group A: ≤2 cm, group B: >2 cm), almost all of the examined operative parameters were more frequently applied to patients of group B: adnexal suspension (100.0% versus 74.1%, resp., NS), suspension of the rectosigmoid (66.7% versus 18.5%, respectively, *P* = 0.012), systematic ureteral dissection (88.9% versus 66.7%, resp., NS), division of uterine vein (33.3% versus 0.0%, resp., *P* = 0.012), colpectomy (33.3% versus 0.0%, resp., *P* = 0.012), and placement of bowel wall reinforcement sutures (33.3% versus 0.0%, resp., *P* = 0.012). On the contrary, excision of pelvic peritoneum was more frequent in group A cases, but not significantly so compared to group B (81.5% versus 77.8%, resp., NS) (Table 4).

Operation times were significantly longer in cases with larger (>2 cm) nodules. Similarly, blood loss was significantly more in cases with larger (>2 cm) and posterolateral nodules (Table 4). We had no intraoperative or immediate postoperative complications. Hospitalization times were not significantly different between groups. All patients included in this study have completed so far at least six months of followup. Complete resolution of CPP (all types) occurred in 75.0% of patients with posterolateral nodules versus 58.3% of those with central nodules and in 74.1% of patients with nodules ≤2 cm versus 55.5% of nodules >2 cm. These differences did not reach statistical significance. During patients' gynecological examination at 6-month followup we recorded presence of any vaginal scarring or induration. Patients with nodule size >2 cm were more likely to present with some degree of scarring compared to those with nodule size ≤2 cm (18.5% versus 66.7%, *P* = 0.012) (Table 4).

## 4. Discussion

Posterior deep infiltrating endometriosis nodules (DIE) represent an operative challenge due to common involvement of vital retroperitoneal structures. Careful dissection is necessary to restore pelvic anatomy and preserve function. Such surgery despite the fact that it may at times become of unpredictable complexity should follow certain rules and well defined operative steps, in order to proceed without serious complications.

The above apply even more to the less experienced surgeon who builds up his/her learning curve in the treatment of such lesions. We conducted this study in an effort to develop a rough guide for the novice operator of DIE, taking into consideration the importance of size, location, and geometry of the nodule, in the process of selecting the appropriate technique to approach each type of lesion. We also feel that by identifying steps absolutely necessary for dissection of the retroperitoneum to advance smoothly, an inexperienced surgeon would benefit, by correcting weaknesses both theoretical and operative and by concentrating on practicing certain operative steps in less complicated cases.

The ENZIAN staging system of DIE was published in 2005. It classifies DIE nodules according to their size and spread along three compartments of the pelvis, vertical, horizontal, and posterior [17]. The truth is that, excluding German speaking countries, the ENZIAN score has been poorly accepted by gynecologists due to the complexity of its documentation and to the absence of significant factors such as pain or infertility incorporated in this system [18, 19]. Nevertheless, we found this system an invaluable aid for the intraoperative development of a strategy to approach these DIE lesions. A simpler yet not descriptive surgical classification is that proposed by Chapron et al., which correlates the type of lesion to the type of procedure necessary to treat each case [12]. In our study we tried to design our surgery by creating two groups of patients: cases with nodules expanding in a predominant fashion laterally and cases with predominantly central nodules, irrespective of the type of their spread along the vertical and posterior axes. In our opinion the key point in attacking both types of posterior nodules is the invariable development of lateral and medial rectovaginal spaces in all DIE cases, a step which the novice should master during his/her learning curve.

Systematic ureteral dissection is a vital step especially when dealing with predominantly lateral (>1 cm, E2b/2bb–E4b/4bb) and larger (>2 cm, all compartments) nodules. Lateral retroperitoneal dissection frequently encounters large vascular structures which represent branches of the internal iliac artery and vein. It is absolutely essential to have a thorough and working knowledge of their anatomy, as it may become necessary to perform extensive vascular dissection with or without division of vessels incorporated in the DIE nodule. The surgeon involved should therefore possess such expertise or be prepared to call for a senior and more experienced assistant.

The technique to manage central nodules is different and has been described in detail [14, 20–23]. We prefer the approach of attacking the vaginal part of the lesion first, leaving the nodule attached to the rectosigmoid, before applying the shaving technique for its excision [14]. The surgeon dealing with central and larger lesions (>2 cm) should have mastered laparoscopic suturing as the application of such skills may become necessary to reinforce or repair the bowel wall, when the shaving technique or discoid excision is performed. In this group of patients we did not have to perform discoid excision. When such or more advanced bowel surgery appears likely, a general surgeon with appropriate skills should be informed in advance, as in many countries such as in Greece, no legal cover exists so far for gynecologists performing intestinal tract procedures. The same applies to ureteral surgery other than ureterolysis.

Very large lesions (>3 cm) represent a different operative platform. In such cases all compartments are frequently involved in a variable manner and designing of surgery in a comprehensive and predictable way, as that described above, may not be of practical value [19]. In such cases, a full preoperative workup should be performed and a multidisciplinary approach is necessary from the start of the procedure to achieve radical excision of the nodule and avoid complications.

Performing routine colpectomy in cases with nodules involving the vaginal wall but sparing the vaginal mucosa appears to be a debatable issue [9, 14, 23, 24]. Recurrence rates of up to 25% have been reported [23], despite the fact that histologically proven endometriosis in the excised vaginal specimen may not exceed 10% [9]. Our technique of removing such nodules without opening the vagina involves a combination of monopolar and bipolar energy. We observed a significant percentage of limited and painless vaginal scaring at 6-month followup in patients treated with the above technique. This finding was more common in patients with central and larger (>2 cm) nodules. Interestingly, in several cases this scarring had subsided considerably at the 12-month followup examination. Whether postoperative asymptomatic vaginal scarring in the noncolpectomized group is associated with an increased risk of recurrence or represents development of fibrosis due to extensive use of electrical energy that tends to subside with time deserves further investigation and longer followup.

In conclusion, surgical steps to excise DIE nodules with laparoscopy may be codified according to lesion size, location, and geometry. Although the ENZIAN scoring system is an important and comprehensive tool to assist in designing surgery, we feel that by simplifying grouping into predominantly lateral and predominantly central lesions, two basic techniques with distinct differences between them arise at least for smaller lesions (≤2 cm). The relatively inexperienced surgeon should try to develop his/her learning curve for each technique by adhering to strict and timely operative steps bearing in mind that for larger or multicompartmental lesions intraoperative adaptation and assistance by more experienced surgeons are of paramount importance to safely conclude the procedure.

## Figures and Tables

**Table 1:** Characteristics of our 36 cases with posterior DIE nodules.

Patient characteristics	Mean (range)	
Age (years)	29.2 (19–36)	
BMI (kg/m^2^)	23.3 (20.2–27.6)	
Gravidity	0.7 (0–3)	
Parity	0.3 (0–3)	

	*N*	%

Previous surgery for endometriosis	4	11.0
Chronic pelvic pain	36	100.0
Dysmenorrhea	32	88.8
Deep dyspareunia	36	100.0
Nonmenstrual pain	23	63.9
Infertility	7	19.4
Ovarian endometriomas	29	80.5
r-AFS classification		
Stage I	1	2.8
Stage II	4	11.1
Stage III	13	36.1
Stage IV	18	50.0

**Table 2:** Distribution of our cases, according to the two main compartments involved, by the DIE nodule. Numbers in brackets represent bilateral involvement (*N* = 36).

ENZIAN system	Less involved compartment			Total
	a	b	c	
Compartment with principal involvement				
a	1*	6 (2)	2	9
b	4	13* (8)	7 (3)	24
c	2	—	1*	3

Total	7	19	10	36

*These cases represent involvement of a single compartment only.

**Table 3:** Intraoperative and postoperative characteristics according to location of the DIE nodule.

Patient characteristics	Central nodules *N* (%)	Lateral nodules *N* (%)	Fisher's exact test *P* value
	*N* = 12	*N* = 24	
Intraoperative characteristics			
Ovarian suspension	7 (58.3)	22 (91.7)	0.029
Rectosigmoid suspension	8 (66.7)	3 (12.5)	0.002
Systematic ureteral dissection	5 (41.7)	21 (87.5)	0.007
Division of uterine vein	0 (0.0)	3 (12.5)	NS
Excision of pelvic peritoneum	6 (50.0)	24 (100.0)	<0.001
Partial colpectomy	3 (25.0)	0 (0.0)	0.031
Rectal wall suturing	3 (25.0)	0 (0.0)	0.031
Mean operation length (mins)	188	179	NS
Mean blood loss (mls)	45	70	<0.001
Postoperative characteristics at 6/12 followup			
Complete resolution of CPP	7 (58.3)	18 (75.0)	NS
Vaginal scarring/induration	5 (41.7)	6 (25.0)	NS

**Table 4:** Intraoperative and postoperative characteristics according to size of the DIE nodule.

Patient characteristics	DIE nodule size ≤ 2 cm *N* (%)	DIE nodule size > 2 cm *N* (%)	Fisher's exact test *P* value
	*N* = 27	*N* = 9	
Intraoperative characteristics			
Ovarian suspension	20 (74.1)	9 (100.0)	NS
Rectosigmoid suspension	5 (18.5)	6 (66.7)	0.012
Systematic ureteral dissection	18 (66.7)	8 (88.9)	NS
Division of uterine vein	0 (0.0)	3 (33.3)	0.012
Excision of pelvic peritoneum	22 (81.5)	8 (88.9)	NS
Partial colpectomy	0 (0.0)	3 (33.3)	0.012
Rectal wall suturing	0 (0.0)	3 (33.3)	0.012
Mean operation length (mins)	124	216	<0.0001
Mean blood loss (mls)	40	85	<0.001
Postoperative characteristics at 6/12 followup			
Complete resolution of CPP	20 (74.0)	5 (55.5)	NS
Vaginal scarring/induration	5 (18.5 )	6 (84.4)	0.012
